# Mandibular Kinematics on an Orthodontic Population Assessed with an Optical Jaw Tracking System: A Comparative Study

**DOI:** 10.3390/dj13050184

**Published:** 2025-04-23

**Authors:** Joana Silva, Ariana Azevedo, Eugénio Martins, Alberto Canabez, Domingo Martin, Conchita Martin

**Affiliations:** 1 Department of Dental Clinical Specialties, Section of Orthodontics, Complutense University of Madrid (UCM), 28040 Madrid, Spain; joanacpr@ucm.es; 2 Faculdade de Medicina Dentária, Universidade do Porto, 4200-393 Porto, Portugalemartins@fmd.up.pt (E.M.); 3 Clinicas Den, 08006 Barcelona, Spain; 4 Clinica Martin Goenaga, 20005 San Sebastián, Spain; 5 BIOCRAN (Craniofacial Biology: Orthodontics and Dentofacial Orthopedics) Research Group, Complutense University of Madrid, 28040 Madrid, Spain

**Keywords:** mandibular kinematics, optical jaw tracking system, orthodontics

## Abstract

**Objective**: To evaluate mandibular kinematics in an orthodontic population using the Modjaw^®^ optical jaw tracking system. **Materials and methods**: A total of 154 orthodontic patients underwent mandibular kinematic analysis using the Modjaw^®^ system. ANB values determined skeletal classification, while dental classification was assessed on digital casts. The Modjaw^®^ records were taken as instructed by the manufacturer, and data collected from the readings included the discrepancy between centric occlusion and maximum intercuspation, maximum opening, Bennett angles, and sagittal condylar guidance. The presence or absence of temporomandibular disorders was determined by the DC-TMD questionnaires. Non-parametric tests and Spearman correlations were applied for the statistical analysis. **Results**: Significant differences in mandibular kinematics were observed between skeletal classes, particularly in CO-MI discrepancies, Bennett angles, and maximum opening (*p* < 0.05). TMD symptoms were associated with higher absolute CO-MI discrepancies but did not significantly alter other kinematic parameters. Weak correlations were found between sagittal condylar guidance and anterior guidance variables. **Conclusions**: Mandibular kinematics differ by skeletal classification, with Class III patients demonstrating distinct patterns. While TMD symptoms impact CO-MI discrepancies, overall mandibular dynamics remain consistent.

## 1. Introduction

The temporomandibular joint (TMJ) is a bilateral synovial joint connecting the temporal bone to the mandible, functioning as a unit. The right and left joints operate together and cannot function independently [1]. Their function is governed by a hinge axis, an imaginary line around which the condyles rotate without translational movement [2]. From this axis, a terminal joint position is determined, crucial in dentistry as a repeatable, recordable alignment with centric relation (CR) [2]. This axis is calculated during pure rotational movement, with its limits determined to be approximately 12 to 15 degrees from maximum intercuspation or 19 to 20 mm at the incisal edges [2].

Mandibular kinematics plays a pivotal role in the evaluation of orofacial functions and understanding dysfunctions associated with various types of malocclusions. Researchers such as Posselt have contributed to the understanding of the border movements of the lower incisors in the sagittal, frontal, and lateral planes [3]. The measurement of mandibular kinematics under dynamic conditions is a valuable tool for evaluating TMJ performance and for enhancing the understanding of the etiology of disorders associated with this joint [4]. Recent epidemiological data report a 34% prevalence of temporomandibular dysfunction (TMD) in the general population, with a 17% comorbidity rate of TMD and bruxism [5]. Moreover, the measurements are used for clinical purposes, including the study of dynamic occlusion in prosthetic restorations, orthodontic treatments, and the screening of TMD [6].

Various techniques have been developed to monitor and record mandibular movement and function, including mechanical tools (e.g., articulators, condylar position indicators, and axiographs), photographic and video recording, radiographic methods, as well as electromagnetic, ultrasonic, and more recently, optical motion capture systems [7,8,9]. Devices such as Modjaw^®^ (Villeurbanne, France) have been developed to analyze and monitor the three-dimensional dynamics of mandibular movements, including opening, closing, laterotrusion, and protrusion [10]. Using optical sensors and morphological mark correlation, the system calculates the patient’s hinge axis with high precision [10,11]. It then uses this algorithm to convert the recorded movements to the CR position, enabling a thorough diagnosis and a holistic treatment plan that considers TMJ function [12,13]. This approach enables a detailed study of mandibular dynamics, providing precise visualization of the condyle’s rotational and translational movements, along with their extent [8,12,14,15]. Therefore, this technology streamlines the recording process and enhances both the accuracy and interpretation of the results [10].

The intermaxillary relationship and the establishment of the correct vertical dimension of occlusion also play a critical role in diagnosing extensive dental treatments and can significantly impact the overall outcome [16].

Some authors have researched the impact of orthodontic treatment and TMD on mandibular kinematics; however, the literature on this topic is controversial, as the available studies are highly heterogeneous [17,18,19,20]. The hypothesis of a relationship between orthodontic treatment and TMD has been largely dismissed; however, there is a lack of literature on the potential impact of different mandibular kinematics on orthodontic treatment and their characteristics within an orthodontic population.

Therefore, the aim of this study was to characterize mandibular kinematics in an orthodontic population. To achieve this, we employed a system that records jaw movements in 3D using optical sensors placed at various points on the mandible.

The main hypothesis of this study was that there were no significant differences in mandibular kinematics across different skeletal or dental classifications within an orthodontic population. The primary goal of this study was to evaluate the determinants of mandibular kinematics in an orthodontic population and compare the results across various skeletal and dental classifications.

## 2. Materials and Methods

This investigation was a monocentric, observational study reported according to the STROBE guidelines [21]. The ethics committee of the institution approved this study (internal code: 22/270-E). All participants gave written informed consent in accordance with the Helsinki Declaration (2024 version).

To estimate the sample size, we selected condylar discrepancy (in millimeters) among Class I, Class II, and Class III patients as one of the main variables. Based on the study by Hidaka et al., the sample size estimation was calculated using G*Power version 3.1.9.7 (Heinrich Heine University Düsseldorf, Germany), assuming a medium effect size (f^2^ = 0.45), a power of 80%, and an alpha level of 0.05 for an ANOVA with three groups. The estimated sample size required per group was 53 participants [22].

### 2.1. Patient Selection

Patients seeking orthodontic treatment at a private practice in Portugal, who had complete initial orthodontic records (including extraoral and intraoral photographs, panoramic and facial profile X-rays, 3D scanner data, and Modjaw^®^ records), were included in the study.

### 2.2. Inclusion Criteria

- Permanent dentition;
- Complete set of orthodontic records.

### 2.3. Exclusion Criteria

- Previous orthodontic or splint treatment;
- Overjet > 8 mm;
- A 100% incisor overlap;
- History of trauma or surgery in the maxillofacial area;
- Systemic conditions affecting the orofacial region;
- Unreliable Modjaw^®^ readings.

### 2.4. TMD Diagnosis

TMD diagnosis was made using the DC-TMD questionnaire from the International Consortium for TMD and Orofacial Pain filled in during orthodontic records appointments [23]. The officially translated and validated version in the language of the participants was used, in accordance with the Consortium’s guidelines for cross-cultural adaptation.

### 2.5. Data Collection and Analysis

One calibrated examiner recorded and analyzed all data, and an intra-rater error analysis was performed. Intraoral models were obtained using the iTero^®^ element Flex scanner (Align Technology, San José, CA, USA) according to the manufacturer’s instructions. The STL files were imported into Modjaw^®^ software (https://modjaw.com/en/, (accessed on 1 March 2025)). The device was calibrated as recommended by the manufacturer, and the tracking devices (Tiara and SMIL’IT) were placed. After confirming the proper adjustment of the tracking devices, the following mandibular movements were recorded with three repetitions: open–close, CR (using Dawson’s bimanual manipulation), protrusion, left and right excursion, speech (numbers 60–70), and chewing.

Using the CR record, a pure rotation was cropped, and the software calculated the patient’s hinge axis. Condylar position graphs were used to measure condylar discrepancy between centric occlusion (CO) and maximum intercuspation (MI). The maximum opening of the open–close movement was determined by measuring the range from the tip of the most extruded upper incisor to the tip of the most extruded lower incisor, plus the overbite (in mm). On protrusive movements, the sagittal condylar guidance angle (in degrees) was determined by the software. The Bennett angles (in degrees) for each TMJ were recorded during lateral excursive movements.

The following data were recorded in a Microsoft Excel^®^ spreadsheet (Microsoft Office, Microsoft Corp., Redmond, WA, USA):

- Presence or absence of TMD signs/symptoms;
- Age;
- Gender;
- Centric occlusion–maximum intercuspation (CO-MI) discrepancy (Modjaw vertical and sagittal, right and left, and transverse) (in mm);
- Overjet (in mm);
- Overbite (in mm);
- Maximum opening (in mm);
- Sagittal condylar guidance (right and left) (in degrees);
- Bennett angles (right and left) (in degrees);
- Angle classification;
- Skeletal classification (ANB angle).

The statistical analysis was performed with the software IBM SPSS, version 29 for Windows (IBM Corp., Armonk, NY, USA).

Normality was tested with the Kolmogorov–Smirnov test that showed that most of the variables did not have normal distribution (*p* < 0.05). Due to the non-normality of the data, non-parametric tests were used. The Kruskal–Wallis test was used for the comparison among angle classifications and among skeletal classifications. Pairwise comparison tests with Bonferroni correction were used when Kruskal–Wallis was significant (*p* < 0.05). The Mann–Whitney test was used for the comparison between patients with and without TMD signs/symptoms. The Spearman correlation coefficient was used for the correlations between continuous variables. The following thresholds were considered to classify the strength of the correlations: 0.00–0.10 negligible, 0.10–0.39 weak, 0.40–0.69 moderate, 0.70–0.89 strong, 0.90–1.00 very strong [24].

A significance level of 5% was considered.

The sample was divided into two groups: under 20 years old (<20) and 20 or over 20 years old (≥20). Student *t*-test for independent variables was performed in order to assess the impact of age on the tested variables.

The statistical analysis was performed with the software IBM SPSS, version 26 for Windows (IBM Corp. Released, 2019).

## 3. Results

The sample included 154 patients, mostly females (72.7%), aged between 11 and 66 years with a mean age of 26.9 years old (SD = 10.5). Regarding angle classification, 37.0% of the patients were Class I, 27.3% Class II, and 35.7% Class III. As for the skeletal classification, 36.4% of the patients were Class I, 42.9% Class II, and 20.8% Class III. Out of the 154 patients, 49 had TMD signs/symptoms, which represented 31.8% of the sample (Table 1).

Descriptive statistics of the variables included in the study are presented in Table 2.

The results for the Student *t*-test for independent variables for individuals under 20 y and over 20 y showed no statistically significant differences for the variables between these subgroups.

Results of the comparison by angle classification are presented in Table 3. Statistically significant differences (*p* ≤ 0.05) were found in the variables CO-MI sagittal distance (right and left), CO-MI vertical right (absolute value), sagittal condylar guidance right, Bennett angle (right and left), maximum opening, overjet, and overbite. Overall, Class III patients had significant lower values in these variables than Class I and Class II patients. The exceptions were CO-MI sagittal right (no differences between Class II and III), CO-MI vertical right—absolute value (no differences between Class I and III), sagittal condylar guidance right (no differences between Class I and III), and Bennett angle right (no differences between Class I and III). There were no significant between Class I and Class II patients in any of the variables (*p* > 0.05), except in the sagittal condylar guidance right (the value was significantly higher in Class II patients than in Class I patients).

As for the comparison by skeletal classification (Table 4) of the CO-MI distances, only the vertical right in absolute value differed significantly (*p* = 0.037): it was significantly lower in Class III than in Class II patients. We also found significant differences (*p* < 0.05) in the variables Bennett angle (right and left), overjet, and overbite. Bennett angles were higher in Class II than in Class I and III patients. Overjet and overbite were significantly lower in Class III patients than in the other patients.

Results in Table 5 show that all the CO-MI distances in absolute values (vertical, sagittal, and transversal) were significantly higher (*p* < 0.05) in the patients with TMD signs/symptoms than in the patients without TMD signs/symptoms. The two groups of patients did not differ significantly in any of the other variables (*p* > 0.05).

Table 6 shows the correlations between a selection of variables. Of the correlations presented, the strongest was found between sagittal condylar guidance right and sagittal condylar guidance left (R_S_ = 0.553, *p* < 0.001). Positive weak correlations were found between the Bennett angles (right and left) and the variables OC-IM sagittal right absolute value (right and left) and sagittal condylar guidance (right and left); the correlation coefficients were significant (*p* < 0.05) but weak (ranging from 0.163 to 0.293). Also positive, significant, but weak correlations were found between Overjet and CR-MI sagittal right (R_S_ = 0.206, *p* = 0.010) and left (R_S_ = 0.209, *p* = 0.009). The correlations between the sagittal condylar guidance (right and left) with the overjet and the overbite were also positive, but weak (R_S_ < 0.180).

## 4. Discussion

This study aimed to analyze mandibular movement patterns in an orthodontic population using the Modjaw^®^ optical tracking system before treatment. By capturing real-time jaw dynamics, it sought to establish a baseline understanding of kinematics, crucial for treatment planning and outcomes. The results provided insights into mandibular function variability, potentially guiding personalized treatment approaches.

Demographic data showed a significant predominance of female patients (72.7%), aligning with literature trends of higher orthodontic demand among women [25]. The sample’s age range was diverse, with 42.9% of patients aged 10–19, aligning with the growth phase when many seek orthodontic correction [26]. This age group is particularly critical, as orthodontic interventions can not only enhance aesthetics and function but also influence craniofacial development [27,28,29].

Our findings show significant differences in mandibular kinematics based on skeletal and dental classifications. Class III patients exhibited lower maximum openings, Bennett angles, and CO-MI discrepancies compared to patients in Classes I and II. These results suggest that Class III patients may experience functional limitations that require careful orthodontic treatment planning. In contrast, no significant differences were found between Class I and Class II patients, indicating that these categories may share similar kinematic characteristics. Class III patients often seek orthodontic treatment later in life, when surgical intervention may be required, thereby increasing their risk for functional disability [30].

When comparing skeletal classifications (Table 4), significant differences were observed in the Bennett angle, overjet, and overbite. Class II patients had higher Bennett angles compared to Classes I and III, while Class III patients exhibited significantly lower values for overjet and overbite (*p* < 0.001). These results suggest that maxillomandibular relationships directly influence mandibular movement dynamics, with a strong impact on orthodontic treatment planning. Although several mandibular kinematic variables showed statistically significant differences between Class III patients and those in Classes I and II, the clinical relevance of these differences should be interpreted with caution. The magnitude of variation, while measurable, may not be substantial enough to directly influence treatment decisions in routine orthodontic cases. These findings primarily highlight functional distinctions that may become more relevant in complex or borderline cases, particularly those involving surgical planning or joint-related concerns. Further studies are needed to determine whether these kinematic differences have a meaningful impact on treatment outcomes or long-term stability.

Interestingly, no studies currently exist in the literature that compare mandibular kinematics prior to orthodontic treatment across Class I, II, or III malocclusions. Further investigations on this topic are therefore warranted.

Additionally, the analysis revealed that the presence of signs and symptoms of TMD was associated with higher absolute values in some measurements, particularly the discrepancy in CO-MI, which is consistent with previous studies [31,32]. Although no substantial impact on overall mandibular dynamics was observed in individuals with TMD, measurable discrepancies in movement patterns suggest that TMD should be considered when planning both initial and ongoing treatment.

Significant correlations were found between the Bennett angles, sagittal condylar guidance, and CO-MI discrepancies, suggesting that condylar displacement may impact mandibular function, with potential clinical implications.

The correlation between the Bennett angle on the left and CO-MI sagittal discrepancy on the right (absolute value) reflects the functional interplay between contralateral condyles during lateral excursions. During mandibular movements, the non-working condyle moves medially and forward, contributing to the Bennett angle observed on the working side. This dynamic interaction highlights the biomechanical nature of TMJ function and supports cross-side correlations as a meaningful approach to analyzing mandibular kinematics. Additionally, the use of absolute values for discrepancies ensures that the magnitude of deviations is captured without bias from directional differences, providing a clearer understanding of compensatory mechanisms. Although Bennett angles are not yet routinely used as a determining factor in orthodontic treatment planning, they may offer valuable insights into TMJ biomechanics. Variations in Bennett angles can reflect asymmetries in condylar movement or joint function, which may be relevant in cases with functional shifts or suspected joint pathology. While our study did not aim to correlate Bennett angle values with specific treatment modalities, recognizing such discrepancies could prompt more detailed joint assessments or influence decisions regarding appliance design, occlusal equilibration, or the need for interdisciplinary collaboration. Future research should investigate whether these measurements can contribute to personalized orthodontic protocols.

Overjet and overbite were also correlated with sagittal condylar guidance, indicating a possible relationship between anterior guidance and condylar function.

The moderate correlation between sagittal condylar guidance on both sides may be explained by the anatomy of the TMJ, as its function is determined by an axis passing through both condyles, which are connected by the mandibular corpus.

However, the weak correlations observed for other tested variables suggest no direct effect of CO-MI discrepancy on mandibular function nor a strong relationship between condylar guidance and dental anterior guidance. This weak correlation aligns with recent studies that challenge the widely accepted relationship between condylar guidance and anterior guidance in restorative dentistry [33,34]. Recent research suggests that condylar guidance may not play a determining role in the occlusal scheme and may be overridden by anterior guidance [33]. This result, however, may be influenced by sample characteristics such as reduced overjet and overbite, which may compromise anterior guidance. Future studies should thoroughly evaluate anterior guidance and include it in eligibility criteria to more accurately assess its impact on condylar guidance.

Finally, significant correlations were found between the Bennett angles and transverse CO-MI discrepancies, indicating an interaction between these variables in mandibular dynamics. This positive correlation suggests that changes in condylar positioning during mandibular movements could have direct implications for mandibular function. These findings highlight the importance of considering Bennett angles in orthodontic planning, as variations in these angles can influence both treatment effectiveness and long-term joint health. This study calls for continued exploration within the orthodontic community regarding the integration of advanced technologies and methodologies to shape more effective treatment paradigms.

Our results led us to reject the null hypothesis as statistically significant differences in mandibular kinematics was observed among different malocclusions.

This study is limited by its cross-sectional design, which does not allow for causal inference, and by the recruitment from a single private clinic, which may affect generalizability. Additionally, although the Modjaw^®^ system provides detailed dynamic measurements, factors such as patient compliance and operator variability may influence the recordings. Future studies should adopt longitudinal, multicenter designs and consider including muscle activity and anterior guidance analysis for a more comprehensive understanding.

## 5. Conclusions

The results revealed statistically significant differences in most mandibular kinematic variables between patients classified as Class I and Class II compared to those classified as Class III. Individuals in Class III exhibited significantly lower values in several key parameters, suggesting that skeletal classification plays a critical role in shaping mandibular kinematic characteristics.

Furthermore, the data indicated that the presence of temporomandibular disorder (TMD) symptoms was associated with higher absolute values in certain measurements, particularly with respect to the CR-MI discrepancy. However, it is important to note that the overall mandibular dynamics in individuals with TMD were not significantly altered, except for the aforementioned discrepancy.

## Figures and Tables

**Table 1:** Sample characteristics.

		*n*	%
*Gender*	Male	42	27.3
	Female	112	72.7
*Age* (years)	10–19	66	42.9
Minimum = 11	20–29	35	22.7
Maximum = 66	30–39	19	12.3
Mean = 26.9	40–49	24	15.6
Standard deviation = 14.0	50–59	7	4.5
	60+	3	1.9
*Angle classification*	Class I	57	37.0
	Class II	42	27.3
	Class III	55	35.7
*Skeletal classification*	Class I	56	36.4
	Class II	66	42.9
	Class III	32	20.8
*TMD signs/symptoms*	No	105	68.2
	Yes	49	31.8

**Table 2:** Descriptive statistics.

	Minimum	Maximum	Mean	SD	CI 95%
CO-MI vertical right	−3.61	4.89	0.12	0.98	−0.04, 0.27
CO-MI vertical left	−3.56	3.53	0.00	0.91	−0.15, 0.14
CO-MI sagittal right	−1.90	2.97	0.00	0.81	−0.12, 0.13
CO-MI sagittal left	−2.70	2.38	0.02	0.76	−0.10, 0.14
CO-MI transversal	−1.36	2.06	0.00	0.49	−0.08, 0.08
CO-MI vertical right—absolute value	0.00	4.89	0.67	0.73	0.55, 0.78
CO-MI vertical left—absolute value	0.00	3.56	0.62	0.66	0.51, 0.72
CO-MI sagittal right—absolute value	0.00	2.97	0.58	0.56	0.49, 0.67
CO-MI sagittal left—absolute value	0.00	2.70	0.54	0.53	0.45, 0.62
CO-MI transversal—absolute value	0.00	2.06	0.33	0.37	0.27, 0.39
Sagittal condylar guidance right	7.00	85.00	49.16	12.11	47.23, 51.09
Sagittal condylar guidance left	19.00	91.00	47.94	11.81	46.06, 49.82
Bennett angle right	−16.00	32.00	6.89	8.18	5.59, 8.19
Bennett angle left	−11.00	31.00	4.59	7.46	3.40, 5.78
Maximum opening	3.00	51.00	35.70	6.21	34.71, 36.69
Overjet	−7.00	10.00	2.75	2.73	2.31, 3.18
Overbite	−6.00	7.50	2.21	2.41	1.83, 2.60

SD—standard deviation; CI 95%—95% confidence interval for the mean.

**Table 3:** Comparison by angle classification.

	Angle Classification			
	Class I (*n* = 57)	Class II (*n* = 42)	Class III (*n* = 55)	*p*
CO-MI vertical right	0.19 (1.13)	−0.02 (1.08)	0.14 (0.71)	0.246
CO-MI vertical left	0.04 (0.81)	0.01 (1.11)	−0.07 (0.85)	0.774
CO-MI sagittal right	0.18 (0.84) ^a^	0.09 (0.85) ^ab^	−0.24 (0.68) ^b^	0.016 *
CO-MI sagittal left	0.14 (0.83) ^a^	0.23 (0.75) ^a^	−0.26 (0.58) ^b^	0.002 **
CO-MI transversal	−0.08 (0.42)	0.02 (0.54)	0.08 (0.53)	0.315
CO-MI vertical right—absolute value	0.74 (0.87) ^ab^	0.81 (0.71) ^a^	0.48 (0.53) ^b^	0.014 *
CO-MI vertical left—absolute value	0.58 (0.55)	0.78 (0.78)	0.53 (0.66)	0.114
CO-MI sagittal right—absolute value	0.62 (0.58)	0.59 (0.61)	0.52 (0.49)	0.615
CO-MI sagittal left—absolute value	0.58 (0.61)	0.58 (0.52)	0.46 (0.44)	0.558
CO-MI transversal—absolute value	0.31 (0.29)	0.34 (0.41)	0.34 (0.41)	0.907
Sagittal condylar guidance right	46.75 (11.23) ^a^	53.71 (9.39) ^b^	48.18 (13.96) ^a^	0.010 **
Sagittal condylar guidance left	47.63 (10.96)	50.33 (10.98)	46.44 (13.13)	0.216
Bennett angle right	7.25 (7.37) ^ab^	9.52 (9.42) ^a^	4.51 (7.37) ^b^	0.027 *
Bennett angle left	4.82 (6.48) ^a^	8.00 (8.28) ^a^	1.75 (6.68) ^b^	<0.001 ***
Maximum opening	37.24 (5.13) ^a^	36.92 (4.83) ^a^	33.18 (7.37) ^b^	0.004 *
Overjet	3.73 (1.77) ^a^	4.88 (1.89) ^a^	0.11 (1.87) ^b^	<0.001 ***
Overbite	2.95 (1.72) ^a^	3.74 (2.26) ^a^	0.28 (1.83) ^b^	<0.001 ***

Results presented as mean (standard deviation); *p*—Kruskal–Wallis test global *p*-value; ^ab^ groups with the same superscript letter did not differ significantly (*p* > 0.05) in pairwise comparisons; * *p* < 0.05; ** *p* < 0.01; *** *p* < 0.001.

**Table 4:** Comparison by skeletal classification.

	Skeletal Classification			
	Class I (*n* = 56)	Class II (*n* = 66)	Class III (*n* = 32)	*p*
CO-MI vertical right	−0.12 (0.97)	0.23 (1.10)	0.29 (0.64)	0.099
CO-MI vertical left	−0.09 (0.70)	0.10 (1.03)	−0.07 (0.96)	0.247
CO-MI sagittal right	0.07 (0.75)	0.09 (0.89)	−0.29 (0.65)	0.085
CO-MI sagittal left	−0.05 (0.67)	0.17 (0.88)	−0.17 (0.54)	0.189
CO-MI transversal	0.01 (0.36)	−0.07 (0.51)	0.15 (0.63)	0.065
CO-MI vertical right—absolute value	0.62 (0.74) ^ab^	0.79 (0.79) ^a^	0.48 (0.51) ^b^	0.037 *
CO-MI vertical left—absolute value	0.54 (0.45)	0.71 (0.75)	0.56 (0.78)	0.217
CO-MI sagittal right—absolute value	0.58 (0.47)	0.61 (0.65)	0.51 (0.49)	0.506
CO-MI sagittal left—absolute value	0.46 (0.48)	0.65 (0.62)	0.43 (0.36)	0.128
CO-MI transversal—absolute value	0.25 (0.26)	0.35 (0.38)	0.42 (0.49)	0.177
Sagittal condylar guidance right	47.50 (11.47)	50.26 (11.22)	49.81 (14.79)	0.305
Sagittal condylar guidance left	48.63 (9.94)	47.97 (11.74)	46.69 (14.89)	0.679
Bennett angle right	5.48 (7.59) ^a^	9.62 (8.56) ^b^	3.72 (6.64) ^a^	0.002 **
Bennett angle left	4.75 (7.69) ^ab^	5.92 (7.68) ^a^	1.56 (5.71) ^b^	0.012 *
Maximum opening	35.18 (7.17)	36.85 (4.98)	34.25 (6.47)	0.163
Overjet	2.66 (2.41) ^a^	4.28 (1.93) ^b^	−0.26 (2.08) ^c^	<0.001 ***
Overbite	2.42 (2.14) ^a^	2.92 (2.54) ^a^	0.37 (1.54) ^b^	<0.001 ***

Results presented as mean (standard deviation); *p*—Kruskal–Wallis test global *p*-value; ^abc^ groups with the same superscript letter did not differ significantly (*p* > 0.05) in pairwise comparisons; * *p* < 0.05; ** *p* < 0.01; *** *p* < 0.001.

**Table 5:** Comparison by TMD signs/symptoms.

	TMD Signs/Symptoms		
	No (*n* = 105)	Yes (*n* = 49)	*p*
CO-MI vertical right	0.07 (0.75)	0.22 (1.36)	0.299
CO-MI vertical left	0.08 (0.73)	−0.18 (1.19)	0.191
CO-MI sagittal right	0.03 (0.69)	−0.05 (1.01)	0.313
CO-MI sagittal left	0.05 (0.64)	−0.04 (0.96)	0.906
CO-MI transversal	−0.07 (0.30)	0.15 (0.74)	0.066
CO-MI vertical right—absolute value	0.52 (0.53)	0.97 (0.97)	0.001 ***
CO-MI vertical left—absolute value	0.51 (0.53)	0.85 (0.85)	0.011 *
CO-MI sagittal right—absolute value	0.50 (0.48)	0.75 (0.67)	0.009 **
CO-MI sagittal left—absolute value	0.46 (0.45)	0.70 (0.65)	0.025 *
CO-MI transversal—absolute value	0.21 (0.22)	0.58 (0.49)	<0.001 ***
Sagittal condylar guidance right	49.30 (10.77)	48.88 (14.70)	0.755
Sagittal condylar guidance left	48.31 (9.89)	47.14 (15.22)	0.664
Bennett angle right	6.07 (7.43)	8.65 (9.42)	0.234
Bennett angle left	4.40 (7.18)	5.00 (8.10)	0.721
Maximum opening	35.76 (6.34)	35.58 (6.01)	0.422
Overjet	2.82 (2.65)	2.59 (2.92)	0.820
Overbite	2.16 (2.42)	2.32 (2.41)	0.709

Results presented as mean (standard deviation); *p*—Mann–Whitney test *p*-value. * *p* < 0.05; ** *p* < 0.01; *** *p* < 0.001.

**Table 6:** Correlations between variables.

	Correlation Coefficient
Bennett angle left vs. CO-MI sagittal right (absolute value)	R_S_ = 0.176, *p* = 0.029 *
Bennett angle right vs. CO-MI sagittal left (absolute value)	R_S_ = 0.216, *p* = 0.007 **
Bennett angle right vs. sagittal condylar guidance right	R_S_ = 0.293, *p* < 0.001 ***
Bennett angle right vs. sagittal condylar guidance left	R_S_ = 0.163, *p* = 0.043 *
Bennett angle left vs. sagittal condylar guidance right	R_S_ = 0.174, *p* = 0.031 *
Bennett angle left vs. sagittal condylar guidance left	R_S_ = 0.190, *p* = 0.018 *
Bennett angle right vs. CO-MI transversal	R_S_ = 0.047, *p* = 0.566
Bennett angle right vs. CO-MI transversal (absolute value)	R_S_ = 0.026, *p* = 0.752
Bennett angle left vs. CO-MI transversal	R_S_ = 0.076, *p* = 0.346
Bennett angle left vs. CO-MI transversal (absolute value)	R_S_ = 0.088, *p* = 0.278
Overjet vs. CO-MI sagittal right	R_S_ = 0.206, *p* = 0.010 **
Overjet vs. CO-MI sagittal left	R_S_ = 0.209, *p* = 0.009 **
Sagittal condylar guidance right vs. sagittal condylar guidance left	R_S_ = 0.553, *p* < 0.001 ***
Sagittal condylar guidance right vs. overjet	R_S_ = 0.075, *p* = 0.355
Sagittal condylar guidance right vs. overbite	R_S_ = 0.157, *p* = 0.052
Sagittal condylar guidance left vs. overjet	R_S_ = 0.119, *p* = 0.140
Sagittal condylar guidance left vs. overbite	R_S_ = 0.178, *p* = 0.027 **
Age vs. CO-MI vertical right	R_S_ = −0.049, *p* = 0.547
Age vs. CO-MI vertical left	R_S_ = 0.009, *p* = 0.911
Age vs. CO-MI sagittal right	R_S_ = −0.130, *p* = 0.107
Age vs. CO-MI sagittal left	R_S_ = −0.148, *p* = 0.066
Age vs. CO-MI transversal	R_S_ = −0.022, *p* = 0.785
Age vs. sagittal condylar guidance right	R_S_ = 0.037, *p* = 0.652
Age vs. sagittal condylar guidance left	R_S_ = 0.062, *p* = 0.443
Age vs. Bennett angle right	R_S_ = −0.114, *p* = 0.160
Age vs. Bennett angle left	R_S_ = −0.220, *p* = 0.006 **
Age vs. maximum opening	R_S_ = −0.071, *p* = 0.385

R_S_—Spearman correlation coefficient. * *p* < 0.05; ** *p* < 0.01; *** *p* < 0.001.

## Data Availability

The data presented in this study are available on request from the corresponding author due to privacy.

## Abbreviations

The following abbreviations are used in this manuscript:

TMJ	Temporomandibular joint
TMD	Temporomandibular dysfunction
CR	Centric relation
CO	Centric occlusion
MI	Maximum intercuspation
MJ	Modjaw
SAG	Sagittal
VERT	Vertical
TRANS	Transverse
L	Left
R	Right
